# Affective lability across psychosis spectrum disorders

**DOI:** 10.1192/j.eurpsy.2020.44

**Published:** 2020-05-06

**Authors:** Margrethe Collier Høegh, Ingrid Melle, Sofie R. Aminoff, Jannicke Fjæra Laskemoen, Camilla Bakkalia Büchmann, Torill Ueland, Trine Vik Lagerberg

**Affiliations:** 1 NORMENT, Centre for Research on Mental Disorders, Division of Mental Health and Addiction, University of Oslo and Oslo University Hospital, Oslo, Norway; 2 Oslo University Hospital, Division of Mental Health and Addiction, Oslo, Norway; 3 Department of Psychology, University of Oslo, Oslo, Norway

**Keywords:** Affective lability, prevalence, psychosis spectrum disorders

## Abstract

**Background.:**

Despite apparent clinical remission, individuals with psychotic disorders often experience significant impairments across functional domains. Thus, there is a need to search beyond management of core symptoms to optimize treatment outcomes. Affective dysregulation is considered a risk factor for poor clinical and functional outcomes in many mental disorders, but research investigating such features in psychosis, particularly in schizophrenia, is limited. We aimed to investigate the level of affective lability (AL) in participants with schizophrenia- and bipolar spectrum disorders (*n* = 222) compared to healthy controls (*n* = 140), as well as clinical correlates of AL in the diagnostic groups.

**Methods.:**

The Affective Lability Scale (ALS-SF) was used to measure total score of AL and subscores covering the domains of anxiety/depression, depression/elation, and anger. An analysis of covariance was performed to compare the ALS-SF total score between groups, correcting for potential confounders, as well as standard multiple regression analyses for diagnosis-specific investigations of the relationship between AL and demographic and clinical features.

**Results.:**

Both the schizophrenia- and bipolar spectrum group had significantly higher ALS-SF total score compared to controls (*p* < 0.001), and no significant differences between the patient groups were found. In the schizophrenia group, current psychotic and depressive symptoms were significantly and independently associated with AL (*p* = 0.012 and *p* = 0.024, respectively).

**Conclusions.:**

The findings indicate that AL is elevated in psychotic disorders and that it transcends diagnostic boundaries. Further research into the causal relationship between psychotic and affective symptoms and AL, as well as its role as a potential therapeutic target in psychosis spectrum disorders, is warranted.

## Introduction

Affective instability (AI) is common in the general population and even more prevalent among persons with mental disorders [1,2]. AI can be defined as rapid oscillations of intense affect with difficulty regulating these oscillations or their behavioral consequences [3], and is considered a central feature of affective dysregulation. The presence of AI in addition to a mental disorder is linked to a more complex and severe illness course and outcome. Research has demonstrated associations with higher rates of compulsory hospital admissions, longer in-patient hospital stays, increased frequency of hospital admissions [4], more frequent suicidal ideation and suicide attempts [5,6], alcohol use disorders (AUD) [7,8], and reduced cognitive and work functioning [9]*.*

The term AI has been used interchangeably with affective lability (AL), mood or emotional instability, and mood swings [3]. The use of different definitions and measurement tools between studies limits the possibility to compare findings across different study populations. As a consequence, it is hard to determine if the negative outcomes associated with AI has consistent implications across diagnoses, or whether the effects pertain to specific mental conditions [10]. In an attempt to clarify these issues, the construct AI has been conceptualized into three core components: the *intensity* of affective responsiveness [11], the ability to *control* affective states [12], and *AL* [3]. Of these components AL, the tendency to experience prominent and unpredictable changes in mood [13], is most commonly investigated and appears to have the highest impact on outcome [10]_._

Individuals with psychotic disorders, schizophrenia-spectrum (SCZ) and bipolar spectrum (BD) disorders in particular, often struggle with psychosocial, vocational, and daily-life functioning even when acute phase affective and psychotic symptoms have diminished [14,15]. Thus, it is necessary to search beyond management of the core clinical symptoms of the disorders to optimize treatment. As this is the case for many mental disorders, the National Institute of Mental Health has proposed a dimensional framework for research, the Research Domain Criteria (RDoC). RDoC aims to improve our limited understanding of the development and maintenance of psychopathology by transcending the boundaries of traditional diagnostic nosology [16]. Consequently, it seeks to combine biological and behavioral components of both normal and abnormal functioning in a singular framework to construct valid phenotypes for mental disorders. Affect regulation, and challenges with such, is a potential mechanism underlying more overt psychopathology, and has recently been suggested as an important new domain within this matrix [17]. As AL has been linked to poor functional outcome in mental disorders, addressing this construct in research could help determine its validity as a clinical treatment target.

Few studies to date have explored AL in psychotic disorders, with the bulk focusing on lability in BD where dysregulation of affect is a core feature. Here, AL belongs to a constellation of symptoms preceding the development of the disorder [18], is present early in the course of illness [19], in manic and mixed episodes [20], but also in periods of euthymia [21]. Hence, AL appears to be both a trait- and state-dependent factor that is associated with poor prognostic outcomes [21,22]. Our research group has previously found relationships between elevated AL and clinical correlates such as AUD, childhood trauma, suicidality, mixed episodes and anxiety, as well as intact executive functioning in BD [7,19,23,24]. In nonaffective psychotic disorders, especially schizophrenia, knowledge concerning the prevalence, distribution, and clinical correlates of AL is scarce [25]. The few existing studies looking explicitly into AL suggest that it is common, and that it may mediate the link between childhood adversity and positive psychotic symptoms [4,10,25]. More broadly, features of affective dysregulation have been associated with both the emergence and persistence of paranoid delusions, auditory hallucinations and other psychotic experiences such as passivity phenomena and thought interference [26–29]. As a consequence, the effects of AL may be of substantial clinical significance in psychotic disorders, but a richer understanding is needed.

Furthermore, there is mounting evidence of considerable overlap between SCZ and BD when it comes to genetic susceptibility and clinical symptomatology [30,31]. A previous study suggests that the level of AL is the same in nonaffective psychotic disorders and BD [10]. To what extent AL is linked to the same sociodemographic factors and clinical symptoms across these diagnostic groups is, however, not known. Also, AL is likely to exist on a continuum from normality to pathology [32], yet few studies looking into AL in severe mental illness have included at-risk populations or healthy controls (HC), with some notable exceptions [10,13,33,34]. This makes it difficult to identify the threshold where AL is so severe that it becomes pathological with need for treatment.

The present study thus seeks to address some of these knowledge gaps concerning AL in psychotic disorders. More specifically, we aim to investigate the distribution and level of AL in individuals with either SCZ or BD and HC. Furthermore, we aim to explore whether there are specific sociodemographic and clinical correlates of AL in the SCZ group, as compared to the BD group.

## Methods

### Participants

We included 222 patients with severe mental disorders, including SCZ (*n* = 88; schizophrenia [*n* = 42], schizophreniform [*n* = 13], schizoaffective [*n* = 8], psychosis Not Otherwise Specified (NOS) [*n* = 25]) and BD (*n* = 134; BD I [*n* = 89], BD II [*n* = 37], and BD NOS [*n* = 8]), and 140 HC who participated in the Thematically Organized Psychosis (TOP) research study at the Norwegian Center for Mental Disorders Research (NORMENT), Oslo University Hospital in Norway. Recruitment to the study is primarily via psychiatric inpatient and outpatient units in a catchment area consisting of all the major hospitals in the Oslo area, and has been ongoing since 2003. HC participants were drawn randomly from the population registers in the Oslo region. To be included in the study, all patients had to meet diagnostic criteria for a Diagnostic and Statistical Manual of Mental Disorders 4th Edition (DSM-IV) diagnosis of schizophrenia- or bipolar spectrum disorder and be able to give informed consent. Before consenting, thorough information about the purpose of the study was given to all participants both orally and in writing, emphasizing the voluntary nature of the study and the opportunity to withdraw at any time. HC were screened with the Primary Care Evaluation of Mental Disorders [35] for a history of physical and mental disorders, ongoing drug or alcohol use and history of severe mental disorders in first-degree relatives. Both patients and HC had to be within the age range of 18–65 years. Exclusion criteria for all participants were intelligence quotient (IQ) below 70, a history of severe head trauma and insufficient understanding of a Scandinavian language. For the current study, only patients and HC who completed the Affective Lability Scale (ALS) [36] were included. A subsample of the current BD group has previously been included in a study of AL and AUD in BD [7]; it is here included in a re-analysis to highlight the differences between SCZ and BD.

The TOP study has been approved by the Regional Committee for Medical Research Ethics and the Norwegian Data Inspectorate and is conducted in line with the Helsinki declaration of 1975, as revised in 2008.

### Clinical assessments

All clinical evaluations were carried out by trained clinical psychologists, psychiatrists, or medical doctors. Diagnoses were based on the Structured Clinical Interview for DSM-IV Axis I disorders, modules A–E. Diagnostic reliability is assessed with regular intervals in the TOP study and has been found to be very good with Cohen’s kappa for diagnosis in the range between 0.92 and 0.99 across different assessment teams. Current psychotic symptoms were assessed with the Positive and Negative Syndrome Scale (PANSS) [37], depressive symptoms with the Inventory of Depressive Symptoms Clinician Rated (IDS-C) [38] for participants in the BD group and the Calgary Depression Scale for Schizophrenia (CDSS) [39] for participants in the SCZ group, and manic symptoms with the Young Mania Rating Scale (YMRS) for participants in the BD group [40]. Internal consistency scores for all of the symptom measures used in the study are presented in Table 1. Lifetime alcohol (AUD) and cannabis (CUD) substance abuse or dependence diagnoses were established according to DSM-IV criteria.

**Table 1.** Internal consistency of the symptom measures

Symptom measure	Cronbach’s alpha
PANSS	0.876
IDS-C	0.795
YMRS	0.767
CDSS	0.828
ALS-SF	0.947

Abbreviations: ALS-SF, Affective Lability Scale-Short Form; CDSS, Calgary Depression Scale for Schizophrenia; IDS-C, Inventory of Depressive Symptoms-Clinician Rated; PANSS, Positive and Negative Syndrome Scale; YMRS, Young Mania Rating Scale.

### Affective lability

We used ALS-SF [41], the short version of the ALS, to capture shifts between normal mood (euthymia) and the domains of anxiety-depression, depression-elation, and anger. Both the ALS and the ALS-SF, which is highly correlated with the original scale, have been found to have good psychometric properties [32,36,42]. The ALS-SF consists of 18 items which are rated on a four-point Likert scale ranging from 0 (“very uncharacteristic of me”) to 3 (“very characteristic of me”). Five of the items refer to shifts in anxiety/depression, eight refer to shifts in depression/elation, and the final five items concern shifts between anger and normal mood. The scale yields a total score of AL (the sum of all item responses divided by 18), as well as subscores for the three affective domains.

### Statistical analyses

All statistical analyses were performed using the Statistical Package for the Social Sciences (SPSS Inc., Chicago, IL, version 24). A significance level of *p* ≤ 0.05 (two-tailed tests) was employed for all tests. Bivariate analyses including a one-way analysis of variance, independent samples *t*-test, and chi-square tests were conducted to compare the groups on demographic and applicable clinical variables, including the level of AL, measured by the ALS-SF total score. For the latter variable, a Tukey’s honestly significant difference (HSD) test was used for post-hoc comparisons, followed by an analysis of covariance to adjust for potential confounders of the relationship between group and the ALS-SF total score. Effect size was calculated using eta square. *Z*-scores were calculated for all of the ALS domains using the means and the standard errors of the mean for the HC as baseline.

Bivariate correlational analyses were then conducted separately for SCZ and BD to investigate relationships between the demographic and clinical variables and the ALS total score. Pearson correlation was used for normally distributed variables and Spearman’s rho for non-normally distributed variables. Demographic variables included gender, age and number of years in education. Clinical variables included duration of illness, current symptoms and medication use. The current symptom variables were chosen in order to examine the relationship between ALS and the core symptoms of SCZ and BD. PANSS positive domain was used to assess psychotic symptoms for both groups, while PANSS negative domain is more prevalent in schizophrenia and was used for SCZ only together with the CDSS. Correspondingly, the IDS-C and the YMRS were chosen for BD. Duration of illness was included to investigate whether the level of AL increases over the course of the illness. Current use of antidepressant (AD) and antipsychotic (AP) medication, in addition to use of mood stabilizers for the BD group, was included since all of these classes of pharmacological agents are known to have stabilizing properties [43]. As associations between AUD and CUD and increased AL in BD have previously been found by researchers from our group [7], these variables were also considered. Lastly, we conducted separate standard multiple linear regression analyses for the ALS-SF total score for SCZ and BD. The clinical and demographic variables shown to be significantly associated with AL in bivariate analyses were entered as independent variables.

## Results

### Demographics and clinical characteristics of the sample

Demographics for SCZ, BD, and HC as well as clinical characteristics for the two diagnostic groups are presented in Table 2. There was a significant difference in gender between the groups, with more women in the BD group compared to HC (*p* = 0.041). In terms of clinical features, the SCZ group had significantly higher total PANSS scores as well as a higher prevalence of AP medication use, a shorter duration of illness and significantly less AUD than the BD group.

**Table 2.** Demographics and clinical characteristics.

	SCZ (n = 88)	BD (n = 134)	HC (n = 140)		
	Mean (SD)	Mean (SD)	Mean (SD)	Statistics	p-value
Age, years	30.3 (9.7)	30.5 (10.3)	32.3 (9.4)	*F* = 1.669, *df* = 2	0.190
Female sex, *n* (%)	41 (46.6)	77 (57.5)	60 (42.9)	*X* ^2^ = 6.154, *df* = 2	**0.046** BD > HC
Education, years, median	14 (3.2)	15 (2.8)	15 (2.0)	*F* = 2.617, *df* = 2	0.074
Duration of illness, years	5.2 (5.2)	10.4 (9.1)		*t* = 4.383, *df* = 216	**0.000**
PANSS—total	57.1 (14.9)	44.4 (8.4)		*t* = 8.282, *df* = 218	**0.000**
IDS-C—total	n.a.	16.5 (10.9)			
CDSS—total	4.39 (4.317)	n.a.			
% > cut-off for moderate depression	34.1^a^	27.6^a^			
YMRS—total	n.a.	3.5 (5.0)			
Lifetime AUD, *n* (%)	6 (6.8)	16 (11.9)		*X* ^2^ = 4.542, *df* = 1	**0.033**
Lifetime CUD, *n* (%)	6 (6.8)	16 (11.9)		*X* ^2^ = 1.561, *df* = 1	0.213
Antipsychotic use, n (%)	72 (81.8)	64 (47.8)		X^2^ = 25.961, df = 1	**0.000**

Abbreviations: AUD, alcohol use disorder; BD, bipolar spectrum disorder; CDSS, Calgary Depression Scale for Schizophrenia; CUD, cannabis use disorder; HC, healthy controls; IDS-C, Inventory of Depressive Symptoms-Clinician Rated; PANSS, Positive and Negative Syndrome Scale; SCZ, schizophrenia spectrum disorder; YMRS, Young Mania Rating Scale.

a CDSS cut-off for moderate depression ≥ 6, IDS-C cut-off for moderate depression ≥ 22.

### ALS-SF scores in the diagnostic groups as compared to HC

**Figure 1.**
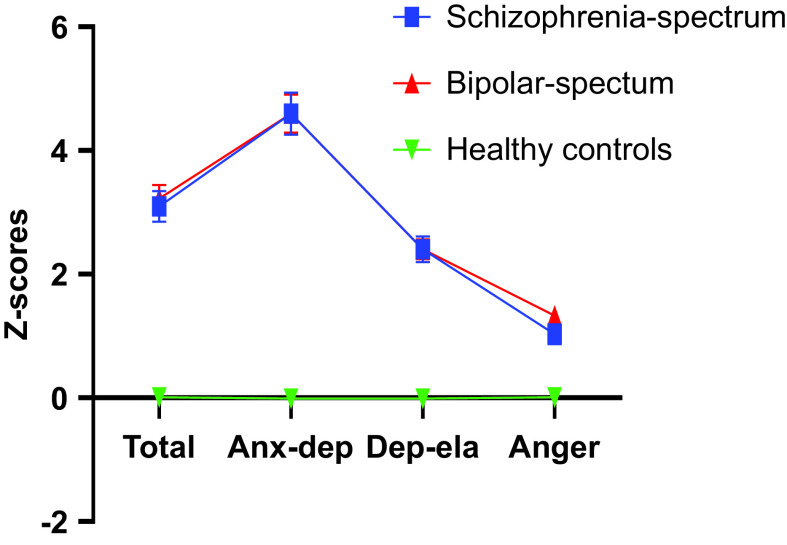
Affective Lability Scale-Short Form score distribution: *Z*-scores for the clinical groups relative to the HC group.

There was a significant difference in the ALS-SF total score between the groups (*F* = 107,258, *p* < 0.001), with a large effect size (eta^2^ = 0.37). Post-hoc comparisons tests showed significantly lower scores for the HC group compared to the SCZ group (*p* < 0.001) and the BD group (*p* < 0.001), but no significant differences between the two diagnostic groups (*p* = 0.903). Correcting for gender, which was differently distributed across groups, did not alter the results. Mean scores for the three groups on all of the ALS-SF subscales are presented in Table 3 and standardized ALS-SF total scores for the clinical groups relative to HC are shown in Figure 1.

**Table 3.** Raw scores for ALS-SF subdomains across the sample

	SCZ	BD	HC
	Mean (SD)	Mean (SD)	Mean (SD)
ALS total	1.16 (0.67)	1.19 (0.73)	0.26 (0.29)
ALS anxiety-depression	1.34 (0.82)	1.32 (0.89)	0.14 (0.26)
ALS depression-elation	1.34 (0.73)	1.33 (0.74)	0.39 (0.39)
ALS anger	0.69 (0.78)	0.85 (0.79)	0.17 (0.51)

Abbreviations: ALS-SF, Affective Lability Scale-Short Form; BD, bipolar spectrum disorder; HC, healthy controls; SCZ, schizophrenia spectrum disorder.

### Associations between ALS-SF total score and demographic and clinical variables in the SCZ group

In the SCZ group, bivariate analyses showed that the ALS-SF was significantly associated with current positive psychotic symptoms and depressive symptoms, but not with gender, number of years in education, age, duration of illness, negative symptoms, AUD, CUD, AD medication use or AP medication use (see Table 4 for correlation coefficients).

**Table 4.** Bivariate correlation coefficients between ALS-SF total score and demographic and clinical variables in the SCZ group

	ALS-SF total	Sex	Years of education	Age	Duration of illness	PANSS P	PANSS N	CDSS	AUD	CUD	AD	AP
ALS-SF total	1.000	*r_s_* = 0.107, *p* = 0.322	*r* = −0.158, *p* = 0.142	*r_s_* = −0.008, *p* = 0.939	*r_s_* = 0.177, *p* = 0.106	***r***_***s***_ **= 0.351, *p* = 0.001**	*r_s_* = 0.033, *p* = 0.759	***r***_***s***_ **= 0.347, *p* = 0.001**	*r_s_* = 0.151, *p* = 0.161	*r_s_* = 0.052, *p* = 0.634	*r_s_* = 0.091, *p* = 0.399	*r_s_* = −0.115, *p* = 0.284

Abbreviations: AD, antidepressant medication; ALS-SF, Affective Lability Scale-Short Form; AP, antipsychotic medication; AUD, alcohol use disorder; CDSS, Calgary Depression Scale for Schizophrenia; CUD, cannabis use disorder; PANSS, Positive and Negative Syndrome Scale; SCZ, schizophrenia spectrum disorder.

In the subsequent multivariate analysis*,* the ALS-SF total score was significantly and independently associated with higher current positive psychotic and depressive symptom scores (model *F* = 7.840, *df* = 2, *p* = 0.001) (Table 5).

**Table 5.** Multiple linear regression analysis on the relationship between ALS-total score and clinical variables in SCZ

				95% CI for B	
Covariates	Beta	t-test	p-value	Lower bound	Upper bound
PANSS positive	0.266	2.582	**0.012**	0.009	0.070
CDSS total	0.237	2.293	**0.024**	0.005	0.069

*R* ^2^ for the final model = 0.157; *N* = 87 due to missing values.

Abbreviations: ALS-SF, Affective Lability Scale; CDSS, Calgary Depression Scale for Schizophrenia; PANSS, Positive and Negative Syndrome Scale; SCZ, schizophrenia spectrum disorder.

### Associations between ALS-SF total score and demographic and clinical variables in the BD group

In the BD group, bivariate analyses showed that the ALS-SF was significantly associated with current depressive symptoms, AUD, AP medication use, use of AD medication, duration of illness and current manic symptoms, but not with gender, age, number of years in education, current positive psychotic symptoms, CUD or use of mood stabilizers (see Table 6 for correlation coefficients).

**Table 6.** Bivariate correlation coefficients between ALS-SF total score and demographic and clinical variables in the BD group

	ALS-SF total	Sex	Years of education	Age	Duration of illness	PANSS P	IDS-C	AUD	CUD	AD	AP	MS	YMRS
ALS-SF total	1.000	*r_s_* = 0.147, *p* = 0.091	*r_s_* = −0.001, *p* = 0.988	*r_s_* = −0.020, *p* = 0.817	***r***_***s***_ **= 0.341, *p* < 0.001**	*r_s_* = 0.138, *p* = 0.113	***r* = 0.421, *p* < 0.001**	***r***_***s***_ **= 0.175, *p* = 0.043**	*r_s_* = 0.056, *p* = 0.523	***r***_***s***_ **= 0.175, *p* = 0.043**	***r***_***s***_ **= −0.347, *p* < 0.001**	*r_s_* = −0.067, *p* = 0.440	***r***_***s***_ **= 0.237, *p* = 0.006**

Abbreviations: AD, antidepressant medication; ALS-SF, Affective Lability Scale-Short Form; AP, antipsychotic medication; AUD, alcohol use disorder; BD, bipolar spectrum disorder; CUD, cannabis use disorder; IDS-C, Inventory of Depressive Symptoms-Clinician Rated; MS, mood stabilizers; PANSS, Positive and Negative Syndrome Scale; YMRS, Young Mania Rating Scale.

In the subsequent multivariate analysis, the ALS-SF total score was significantly and independently associated with higher current depressive symptom scores and with having an AUD. Also, individuals not using AP medication had higher scores compared to those with AP medication use. The final model was significant (*F* = 8.936, *df* = 6, *p* < 0.001) (Table 7).

**Table 7.** Multiple linear regression analysis on the relationship between ALS-total score and demographic and clinical variables in BD

				95% CI for B	
Covariates	Beta	t-test	p-value	Lower bound	Upper bound
IDS-C total	0.331	4.151	**0.000**	0.012	0.033
YMRS total	0.020	0.262	0.793	−0.020	0.026
Duration of illness	0.124	1.543	0.126	−0.003	0.023
AP use	−0.261	−3.263	**0.001**	−0.614	−0.150
AD use	0.068	0.866	0.388	−0.139	−354
AUD	0.155	2.041	**0.043**	0.009	0.616

*R* ^2^ for the final model = 0.303; *N* = 128 due to missing values.

Abbreviations: AD, antidepressant medication, ALS, Affective Lability Scale; AP, antipsychotic medication, AUD, lifetime alcohol use disorder; BD, bipolar spectrum disorder; IDS-C, Inventory of Depressive Symptoms—Clinician Rated; YMRS, Young Mania Rating Scale.

## Discussion

To the best of our knowledge, this is the largest study to date exploring AL across a clinical sample of patients with SCZ and BD disorders compared to HC from the same catchment area. Our main findings were that the patients had significantly higher levels of AL compared to HC, but that there were no significant differences between the SCZ and BD groups with respect to the total level of AL. In BD, where affective dysregulation is inherent to the disorder itself, one would expect elevated AL, but our results indicate that AL is an equally relevant clinical feature in SCZ. This observation calls for further attention to AL both within research and clinical care, and the current study also adds to the knowledge of AL in psychotic disorders by investigating its relationship with clinical characteristics.

We found that depression was significantly associated with elevated AL in both diagnostic groups. Depressive symptoms are troublesome in their own right, but our findings also demonstrate that they are linked to increased lability in affect, which may further add to the illness burden. As the ALS-SF contains several items pertaining to depressive experiences, one might suspect that the observed association is due to a phenomenological overlap. However, the depressive experiences entailed in the ALS-SF refer to rapid *switches* between depressive and other emotional states such as normal mood or anxiety, not depressive symptoms per se. Depression in schizophrenia has long been a diagnostic conundrum, with accumulating evidence of it being intrinsic to the illness rather than a comorbidity [44]. Yet, despite its prevalence and prominence, there are limited studies investigating treatment alternatives for depression in schizophrenia. Although the causal directions are unknown, targeting AL and other features of affective dysregulation could potentially provide a buffer against depression [27]. Conversely, AL may also be a facet or consequence of depression. As we state in the introduction, AL has been found in periods of euthymia in BD [21], indicating that there are features of AL that are more “trait-like” and not simply a function of elevation in symptom levels. In schizophrenia, the prevalence of AL in non-symptomatic patients is not known and needs to be investigated further. However, the clinical symptom scores of our SCZ group indicate that the majority is in the “mildly ill” category [45], and yet we still found a statistically significant difference in AL between patients and HC. We tentatively interpret this in support of the claim that AL is a risk factor for psychopathology, and that intervention efforts are needed. Also, a relationship between AL and increased positive psychotic symptoms was found in the SCZ group. Clarifying this interplay is important: do psychotic symptoms increase AL or does AL increase the risk for reality distortion? The latter would be in line with the notion of an affective pathway to psychosis [46]. To investigate these relationships, longitudinal studies with frequent assessments of AL and psychotic and depressive symptoms in parallel are necessary.

We have previously explored clinical correlates of AL in individuals with BD [7,23,24]. In the current study, we also investigated the relationship between AL and the most commonly used psychopharmacological agents and found that AL was lower in individuals with BD using AP medication. Our results support those of Cipriani et al. [43] indicating that AP medication has good mood-stabilizing properties in BD and extend the findings to a group of BD patients with fairly low levels of depressive and manic symptoms. The observed association was, however, not present in the SCZ group. This may suggest that AP medication does not have the same mood-stabilizing effect in SCZ, but could also be a statistical ceiling-effect since the majority of the SCZ group used such medication. The association between AUD and AL in the BD group, a link we have shown previously [7], was not found in the SCZ group. This could be a type II error, as only six individuals in the SCZ group had AUD. Taken together, the findings suggest that although the level of AL was equally high across diagnoses in our sample, the paths leading to this elevation may be diagnosis-specific.

There are no proposed or validated cut-off scores for evaluating the severity of AL. Previously, mean ALS-SF total and subscale scores in the range of 0.38–0.86 for HC, 1.16–1.66 for patients with BD and 1.25–1.65 for patients with nonaffective psychosis respectively, have been found by Marwaha et al. [10], which correspond well with our results. Future studies should aim to establish severity cut-off values for the ALS-SF as this would be useful both for clinical purposes and in research. From a clinical perspective, exploring the implications of AL in psychotic disorders may be fruitful since affective disturbances are considered burdensome and highly prioritized as treatment targets by service users, even more so than positive psychotic symptoms [47,48]. Focusing on aspects of affective dysregulation might consequently lead to increased satisfaction with, and corresponding adherence to, treatment.

### Limitations and strengths

Our findings must be interpreted in light of some limitations. The ALS-SF is a self-report instrument which makes it vulnerable to recall- and response bias. Also, we cannot make causal attributions about the associations between the clinical variables and elevated AL due to the cross-sectional nature of the study. Furthermore, an investigation of potential differences in AL between the different diagnoses included in the SCZ and BD groups would have been informative, but this was not possible due to small sample sizes. The study also has several strengths; it is the largest study to date looking at AL in a transdiagnostic, representative, well-characterized and relatively young sample of individuals with psychotic disorders, as well as HC.

### Conclusions

Our results illustrate that AL is markedly elevated in psychotic disorders and that it transcends diagnostic boundaries. In the SCZ group, AL was associated with higher levels of current depressive and positive psychotic symptoms. In BD, in addition to previously known relationships to AUD and depressive symptoms, AL was less prominent in individuals using AP medication. Further research is needed to establish whether elevated AL increases affective and/or psychotic symptom load in these patient groups or vice-versa. Nevertheless, our findings indicate that AL may be a relevant therapeutic target in psychotic disorders and that it is warranted to investigate how strategies aiming to promote affective stability, such as emotion regulation skills training, could be integrated in the treatment of these patient populations.

## Data Availability

The data that support the findings of this study will be made available upon request.
